# Intermarriages

**Published:** 1870-10

**Authors:** 


### INTERMARRIAGES.

Observation has proven conclusively to our mind that consanguineous marriages are only injurious by reason of hereditary imperfections. For instance, if a robust, healthful and handsome young man marry his own cousin, equally healthy and handsome, their children will most likely inherit all the characteristics of the parents. But should a feeble, deformed, or sickly young man marry his cousin, equally enfeebled in health, their offspring will probably be sickly and deformed.

Should an uncle marry his neice, the chances for healthy offspring are not so good, by reason of the disproportion of the ages of the parents.

There is a celebrated family of jews residing in Amsterdam, who have intermarried for centuries, yet their physique is superb. But cousins should never marry, unless both parties are in most perfect form and health.

If our young friends can gather any comfort from our remarks upon this subject, they are welcome to the happiness afforded.

MILK AS A PREVENTIVE OF LEAD POISONING.—M. Didierjean, a red lead manufacturer in France, states that he tried every possible way to keep his workmen in good health, but did not wholly succeed in preventing lead colics until by mere accident he found out that two of his men were never affected in that way. Inquiry brought out the fact that these men regularly took milk as a drink with their meals. He was thus led to try the experiment of making the use of milk (a litre a day) compulsory with the workmen, and he has succeeded by this means in keeping all of them free from any symtom of lead disease for the past eighteen months. The absolute correctness of this statement is confirmed by good authority. The remedy is a simple one, surely, and it ought to be thoroughly tested by every person exposed to the danger of lead poisoning.

VINE CULTURE IN FRANCE.—According to the Bulletin of the Scientific Association of France, the vine occupies in that country 2,500,000 hectares, or 6,175,000 acres,—the sixteenth part of all the land capable of cultivation. The gross produce amounts to 1,500,000,000 francs, or about $300,000,000 in gold. This industry gives employment to six million men, women, and children, nearly two million merchants, agents, etc.

